# Reduction Patterns of Acute Schistosomiasis in the People's Republic of China

**DOI:** 10.1371/journal.pntd.0002849

**Published:** 2014-05-08

**Authors:** Shi-Zhu Li, Hao Zheng, Eniola Michael Abe, Kun Yang, Robert Bergquist, Ying-Jun Qian, Li-Juan Zhang, Zhi-Min Xu, Jing Xu, Jia-Gang Guo, Ning Xiao, Xiao-Nong Zhou

**Affiliations:** 1 National Institute of Parasitic Diseases, Chinese Center for Disease Control and Prevention, Shanghai, People's Republic of China; 2 Key Laboratory of Parasite and Vector Biology, Ministry of Health; WHO Collaborating Center for Malaria, Schistosomiasis and Filariasis, Shanghai, People's Republic of China; 3 Department of Zoology, Federal University Lafia, Lafia, Nasarawa State, Nigeria; 4 Jiangsu Institute of Parasitic Diseases, Wuxi, People's Republic of China; 5 Ingerod 407, Brastad, Sweden; University of Nottingham, United Kingdom

## Abstract

**Background:**

Despite significant, steady progress in schistosomiasis control in the People's Republic of China over the past 50 years, available data suggest that the disease has re-emerged with several outbreaks of acute infections in the early new century. In response, a new integrated strategy was introduced.

**Methods:**

This retrospective study was conducted between Jan 2005 and Dec 2012, to explore the effectiveness of a new integrated control strategy that was implemented by the national control program since 2004.

**Results:**

A total of 1,047 acute cases were recorded between 2005 and 2012, with an annual reduction in prevalence of 97.7%. The proportion of imported cases of schistosomiasis was higher in 2011 and 2012. Nine clusters of acute infections were detected by spatio-temporal analysis between June and November, indicating that the high risk areas located in the lake and marshland regions.

**Conclusion:**

This study shows that the new integrated strategy has played a key role in reducing the morbidity of schistosomiasis in the People's Republic of China.

## Author Summary

A retrospective study on the incidence of acute schistosomiasis in the People's Republic of China (P.R. China) was performed, in order to assess the new integrated control strategy that was implemented through the national control program from 2005 to 2012. The lake and marshland regions have been identified as high risk areas as shown by the nine spatio-temporal clusters that we found in the transmission period between June and November each year. When a total of 1,047 reported cases of acute schistosomiasis were analyzed, a reduction in prevalence of 97.7% between 2005 and 2012 was found. In contrast, imported cases of acute schistosomiasis increased between 2011 and 2012. These findings support the approach and effectiveness of the new integrated strategy in the reduction of schistosomiasis morbidity.

## Introduction

**Figure 1:**
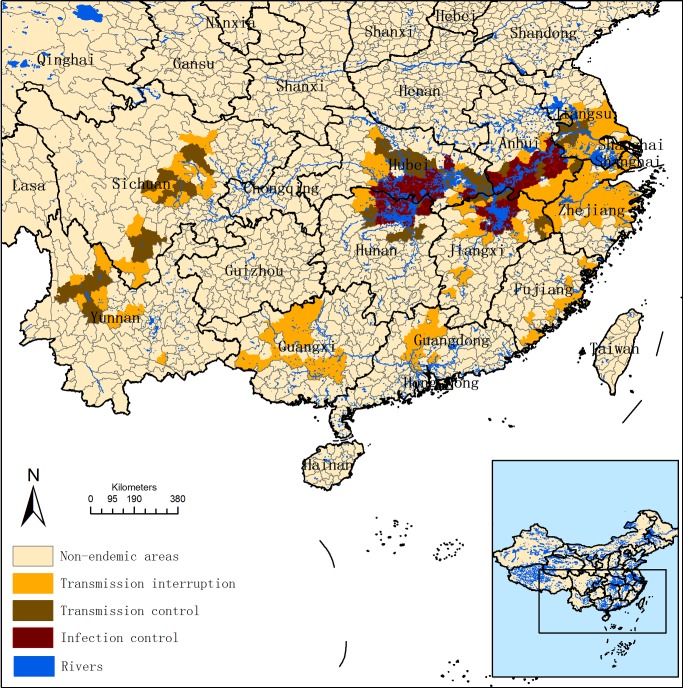
Schistosomiasis distribution in China in 2011.

Schistosomiasis japonica, caused by *Schistosoma japonicum*, is a serious parasitic zoonosis threatening millions of people in the Southeast Asia, including the Peoples' Republic of China (P.R. China) [1]. Despite the great achievements made during the last six decades in the control of the disease [2], it remains a public health concern in P.R. China [3]. Changes in the ecosystem due to environmental degradation and infrastructural development contributed to the resurgence of schistosomiasis in the early 21^st^ century. The National Schistosomiasis Control Program was established already in the early 1950s [3] and the disease was recently given top priority together with HIV/AIDS and tuberculosis [4]. The development of an effective schistosomiasis control program gained in importance and the State Council issued the national medium and long-term strategic work plan in 2004 for schistosomiasis control to be achieved by 2015. The current strategy is focused on control of infection sources, a shift from the earlier strategy of morbidity control [5], [6], in order to strengthen the implementation of integrated measures aiming to reduce the transmission of *S. japonicum* from cattle and humans to snails [7]. The control strategy includes the following main interventions: (i) replacement of bovines and most water buffalo by tractors for agricultural activities [8]; (ii) rearing livestock in pens and forbidding them to pasture in marshlands where snail habitats exists [9]; (iii) recycling excreta from humans and domestic animals to produce methane for cooking [10]; (iv) requiring fishermen to use containers to prevent excreta from being released into the water in Poyang Lake and Yangtze River area [11], (v) improving the environment in high risk areas [12]; and (vi) implementing other routine health-control measures, such as snail survey and elimination, regular surveys and treatments, and health education [13], [14]. This work plan has been boosted by joint efforts from both the central and local governments to produce an effective control program. By the end of 2008 the medium term goal was achieved, i.e., infection controlled with a prevalence rate less than 5% without any outbreak of acute schistosomiasis in all endemic areas, and achievement of transmission control with a prevalence rate less than 1% both in humans and reservoir hosts in the mountainous areas (Sichuan and Yunnan provinces) [15]. In addition, Jiangsu province, located in the lower part of the Yangtze River, attained the status of transmission control in 2010 [11], [16]. Of the 454 counties earlier reported endemic for acute schistosomiasis, 274 (60%) attained the status of transmission interruption, and 103 (23%) achieve transmission control with the remaining 77 counties (17%) for reaching infection control by the end of 2011 [17] (Figure 1).

Due to the complex nature of schistosomiasis and its sometimes irregular distribution, the disease still remains an important public health concern in P.R. China [15], [18]. Despite strong progress, control activities remain arduous with efforts on the schistosomiasis control program continuing to ensure total elimination of transmission [11], [19]. It is now essential to further identify the critical epidemiological factors, monitor potential risk areas and provide technical guidance for surveillance and response during the continuing shift in strategy from morbidity control to focus on transmission. This change requires close oversight of the infection status, not only of humans but also of livestock and the intermediate *Oncomelania* snail host [20].

Since acute schistosomiasis is a highly sensitive indicator in the process of monitoring and evaluating the progress of Schistosomiasis control [4], it should be the first step in the ongoing major transformation of the control program [21]. However, we are yet to understand fully the role of this indicator in strategy shift from control to elimination. This retrospective study seeks to explore the effectiveness of a new integrated control strategy that was implemented through the national control program, based on reported cases from 2005 to 2012 in P.R. China.

## Methods

### 1. Data collection

Data of cases and outbreaks were extracted from web-based National Notifiable Infectious Diseases Reporting Information System (NIDRIS) with a timeframe of 2005–2012. There are 38 notifiable diseases (including schistosomiasis) in P.R. China and all cases are compulsorily reported through NIDRIS according to the National Regulation on the Control of Communicable Diseases since 2005 [22]. Therefore, as soon as an acute schistosomiasis case is diagnosed, it will be reported through the NIDRIS system within 24 hours, including information such as gender, occupation, age, residential address, date of accident or diagnosis, type of diagnosis, infection or outbreak site, etc. All reported acute cases were diagnosed in accordance with the document of the National Criterion on the Schistosomiasis Diagnosis (WS 261-2006 edition) [23]. Briefly, the acute case is considered due to the patient who has: (i) history of water contact in the endemic areas of schistosomiasis from last four weeks to three months, (ii) symptoms of fever, hepatomegaly and peripheral eosinophilia, and (iii) schistosoma eggs found from faeces. Outbreaks are determined in accordance with the criteria of Response and Management Scheme for Schistosomiasis Outbreaks [8] and confirmed by experts from the National Institute of Parasitic Diseases, Chinese Center for Disease Control and Prevention [24]. An imported case of acute schistosomiasis is recorded when confirmed to have been infected outside the reported region, e.g. county or province.

Both active and passive surveillance covers personal and clinical information, including patient's name, age, gender, address, diagnosis, infection locality, the type of water contact, and the type of endemic area which has been classified and defined in the nationwide schistosomiasis survey conducted in 2004 [25]. The active surveillances were mainly carried out in 82 national surveillance sites covering eight provinces, with snail detection in the Spring and Autumn every year and samples for case detection taken annually in the Autumn. The passive surveillance was carried out in all clinics or hospitals at village, township and county levels.

### 2. Statistical analysis

A database was established by double input into Microsoft Excel 2003, and all data in the established database were analyzed using SPSS v17.0 [26]. The chi-square test was used to explore associations between the infection status and age, occupation and sex.

### 3. Spatiotemporal clustering analysis

The space-specific database was extracted from the database (including imported cases) with the criterion of (i) cases without identifiable infection location, and (ii) infected locations without clear coordinate information. Then the secondary space-time database was established with the county center-of-mass coordinate extracted from the national county electronic map (1∶250 000) in ArcGIS 9.3 (ESRI, Redlands, CA, USA). The space-time clustering was performed by Retrospective Space-Time analysis using discrete Poisson model in SaT Scan V9.1.1 [27]. The time aggregation was specified as 7 days, which was same as the period of weekly report of acute infection with *S. japonicum*, followed by Monte Carlo reiteration statistics 999 times. ArcGIS 9.3 was used to visualize the space-time cluster regions in map.

## Results

### 1. General status

A total of 1,047 cases were reported as acute schistosomiasis through the NIDRIS from 2005 to 2012. Among these, 850 (81.18%) were confirmed by stool examination, 197 (18.82%) were clinically diagnosed, and 85 (8.12%) recorded as imported cases. After a peak of 1,114 cases in 2003, a significant reduction in the number of acute schistosomiasis cases presented from 2005 to 2012. A 97.7% reduction was recorded as the yearly report showed a decline from 564 to 13, and the incidence rates per 100,000 population ranging from 0.331 to 0.007, dropped by 97.8%. Although there are two micro-rebounds in 2009 and 2012, the lowest was only 3 cases in 2011 (Table 1). The proportion of imported cases increased sharply after 2009, especially in 2011 and all three reported cases were identified as imported cases (Table 1). Only five outbreaks were reported in Hubei province and one in Sichuan province in 2005, but none was recorded in subsequent years.

**Table 1:** Composition of acute schistosomiasis cases from 2005 to 2012.

Year	Reported Cases	Incidence rate (1/100 000)	Diagnosed Case (%)	Clinically Case (%)	Imported cases (%)	Outbreaks
2005	564	0.331	460(81.56)	104(18.44)	35(6.21)	6
2006	207	0.121	161(77.78)	46(22.22)	10(4.83)	0
2007	83	0.048	71(85.54)	12(14.46)	9(10.84)	0
2008	57	0.032	46(80.70)	11(19.3)	6(10.53)	0
2009	77	0.043	65(84.41)	12(15.59)	9(11.7)	0
2010	43	0.024	35(81.40)	8(18.60)	3(6.98)	0
2011	3	0.002	3(100)	-	3(100)	0
2012	13	0.007	9(69.23)	4(30.77)	10(76.9)	0
Total	1047	-	850(81.18)	197(18.82)	85(8.12)	6

### 2. Distribution

#### 2.1 Temporal distribution

**Figure 2:**
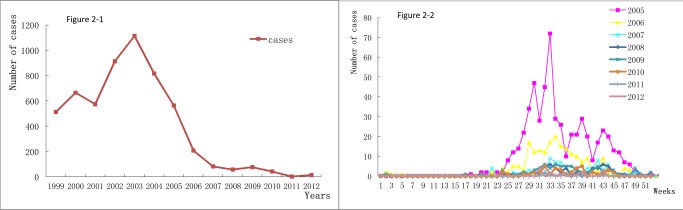
Annual and weekly trends of acute schistosomiasis cases from 2005–2012.

The annual number of reported cases declined significantly from 2005 to 2008 (89.89% decrease), then reached a peak with the lowest in year 2011 (n = 3, 0.002 per 100,000 population) and two micro-rebounds in 2009 (n = 77, 0.043 per 100,000 population) and 2012 (n = 13, 0.007 per 100,000 population) (Figure 2). The weekly-based reported cases indicate that the first annual reported case of acute schistosomiasis occurred on January 5, 2009 (reported by Guangxi Zhuang Autonomous Region), while the last annual reported case was on October 28, 2009 (reported by Jiangxi province). The number of the reported cases increased in the 16th or 17^th^ week and cases are at peak from the 30th to 39th week of each year, after which the number significantly dropped. No significant number of cases was reported from 2007 to 2012, but with a small peak during the 33rd and 43rd week (Figure 2).

#### 2.2 Spatial distribution

A total of 989 (94.46%) cases were reported in the lake and marshland regions (Hunan, Hubei, Jiangxi, Anhui and Jiangsu provinces), while 55 (5.25%) cases in the mountainous regions (Sichuan and Yunnan province) and 3 (0.29%) cases of *S. haematobium* imported from Ethiopia were reported between 2005 and 2012. The pattern in spatial distribution of acute cases did not change significantly from 2005 to 2012, while the acute cases majorly occurred in the lake and marshland regions (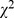 = 20.788, *P*<0.001). There were no reported cases of acute schistosomiasis in the mountainous region from 2008. Despite a few imported cases in non-endemic areas or transmission-interrupted areas every year, the lake and marshland regions were identified as endemic-central areas according to epidemiological investigations (Table 2).

**Table 2:** Composition of acute schistosomiasis cases in different regions from 2005 to 2012.

Region	2005 No. (%)	2006 No. (%)	2007 No. (%)	2008 No. (%)	2009 No. (%)	2010 No. (%)	2011 No. (%)	2012 No. (%)	Total No. (%)
Lake region	515 (91.31)	203 (98.07)	81 (97.59)	56(98.25)	75(97.4)	43(100)	3(100)	13(100)	989(94.46)
Mountainous region	49 (8.7)	4 (1.9)	2 (2.4)	0	0	0	0	0	55(5.25)
Imported from Africa	0	0	0	1(1.75)*	2(2.6)*	0	0	0	3(0.29)
Total	564	207	83	57	77	43	3	13	1047

*cases from Ethiopia infected with *S. haematobium*.

Hubei province had the highest number of cases with 25.31% in endemicity level, followed by Anhui (24.26%), Jiangxi (22.25%), Hunan (17.86%) and Jiangsu (2.87%) provinces, respectively, as reported by NDRIS. This indicates that the distribution of acute schistosomiasis is focused in the lake and marshland regions, though no case of schistosomiasis was reported in Jiangsu province in 2011 as shown in Table 3.

**Table 3:** Number of acute schistosomiasis cases and outbreaks reported by provinces from 2005 to 2012.

Province	2005			2006			2007			2008			2009			2010			2011			2012			Total		
	TC	IC	OB	TC	IC	OB	TC	IC	OB	TC	IC	OB	TC	IC	OB	TC	IC	OB	TC	IC	OB	TC	IC	OB	TC	IC	OB
Hunan	81	4	0	36	0	0	15	1	0	15	0	0	24	0	0	18	0	0	0	0	0	0	0	0	189	5	0
Hubei	165	6	5	52	3	0	26	0	0	8	1	0	9	1	0	3	0	0	0	0	0	0	0	0	263	11	5
Jiangxi	137	1	0	57	0	0	19	1	0	9	0	0	13	0	0	2	0	0	0	0	0	3	1	0	240	3	0
Anhui	121	2	0	55	0	0	21	0	0	21	0	0	25	4	0	17	0	0	0	0	0	5	4	0	265	10	0
Jiangsu	11	9	0	3	4	0	0	1	0	3	1	0	2	0	0	2	2	0	0	0	0	3	3	0	24	20	0
Sichuan	34	0	1	2	0	0	1	0	0	0	1	0	0	0	0	0	0	0	0	0	0	0	0	0	37	1	1
Yunnan	15	0	0	2	0	0	1	0	0	0	0	0	0	0	0	0	0	0	0	0	0	0	0	0	18	0	0
Shanghai	0	0	0	0	1	0	0	1	0	0	0	0	0	0	0	0	0	0	1	1	0	0	0	0	1	3	0
Zhejiang	0	3	0	0	1	0	0	1	0	0	2	0	1	1	0	0	0	0	2	2	0	1	1	0	4	11	0
Fujian	0	1	0	0	1	0	0	3	0	0	0	0	0	0	0	0	0	0	0	0	0	0	0	0	0	5	0
Guangdong	0	2	0	0	0	0	0	1	0	0	0	0	0	0	0	0	0	0	0	0	0	0	0	0	0	3	0
Guangxi	0	1	0	0	0	0	0	0	0	0	0	0	1	1	0	0	0	0	0	0	0	0	0	0	1	2	0
Guizhou	0	5	0	0	0	0	0	0	0	0	0	0	0	0	0	0	0	0	0	0	0	0	0	0	0	5	0
Henan	0	1	0	0	0	0	0	0	0	0	0	0	0	0	0	0	0	0	0	0	0	0	0	0	0	1	0
Beijing	0	0	0	0	0	0	0	0	0	1	1*	0	2*	2	0	1	1	0	0	0	0	0	0	0	4	4	0
Chongqing	0	0	0	0	0	0	0	0	0	0	0	0	0	0	0	0	0	0	0	0	0	1	1	0	1	1	0
Total	564	35	6	207	10	0	83	9	0	57	6	0	77	9	0	43	3	0	3	3	0	13	10	0	1047	85	6

TC = Total number of acute cases; IC = Imported cases; OB = Outbreaks.

*cases from Ethiopia.

#### 2.3 Population distribution

**Figure 3:**
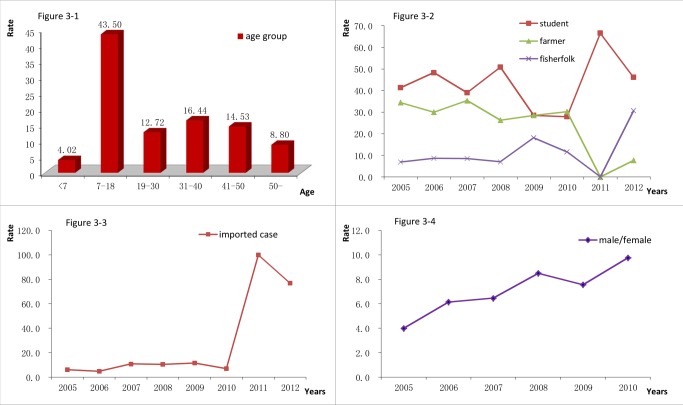
Population distribution of acute schistosomiasis cases from 2005 to 2012 (Figure. 3-1: age distribution, Figure 3-2: occupational distribution, Figure 3-3: annual distribution of imported cases, Figure 3-4: annual ratio of male/female from 2005 to 2010).

Acute schistosomiasis cases were found across the different age groups, most of which were in the 7–18 age group (43.50%), followed by the 31–40 age group (16.44%), the 41–50 age group (14.53%), 19–30 age group (12.72%), above 50 (8.80%) and pre-school age group (1–6 years old, 4.02%). The youngest age in which infection was reported was in a 1-year old child, while the oldest was 83 years old (Figure 3-1). The proportion of acute cases in different age groups showed no significant change from 2005 to 2012(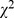 = 43.325, *P* = 0.158), except a decline in the 7–18 years age group in 2009 (28.57%) and 2010 (32.56%). Considering school age groups, most of them were primary school students (56.48%), followed by the junior high school group (32.53%) and senior high school group (10.99%).

With regard to occupations, most acute cases of infection were recorded in school-aged children (n = 436, 41.68%) and farmers (n = 336, 32.2%), followed by fishermen (n = 91, 8.7%), workers (n = 44, 4.21%), pre-school children (n = 38, 3.60%), business workers (n = 20, 1.91%), public officers (n = 10, 1.0%) and others (n = 52, 4.97%). Based on eight years of investigation acute cases of schistosomiasis were always found to be higher in students (range from 28.57% to 66.67%) and farmers (range from 26.32% to 35.37%) each year. The proportion of students' cases in 2009 and 2010 decreased compared with other years, and the proportion of farmers reduced in 2012, did not present a significant difference in the total ratio of these two occupations (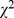 = 9.902, *P* = 0.190). However, the constituent ratio of students, farmers and fishermen showed a significant difference compared with other occupations (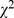 = 32.495, P = 0.005) (Figure 3-2).

**Figure 4:**
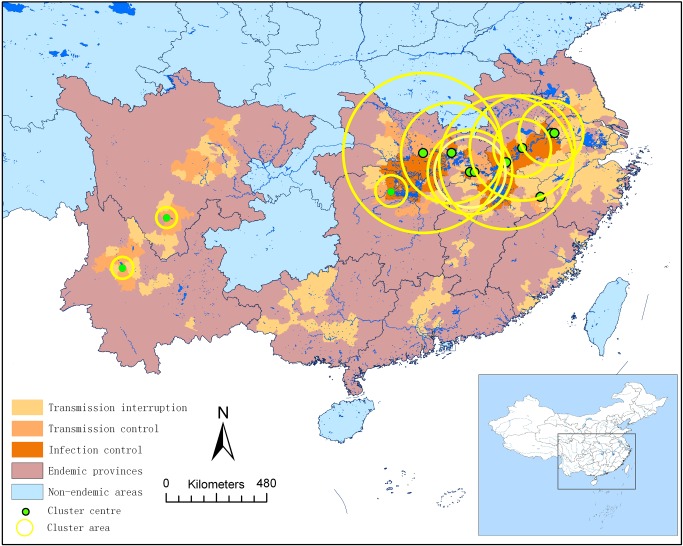
Spatial clustering of acute cases infected with *S. japonicum* from 2005 to 2012.

The proportion of imported cases varied from 6.21% to 100% between 2005 and 2012. It showed a significant change since 2010 to 2012 (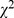 = 38.571, *P*<0.001) comparing with the results of 2009 and before, when the case proportions ascended to 100% (2011) and 76.92% (2012) sharply (Figure 3-3). The proportion of males/females remained higher (875/142) yearly with an ascending trend from 2005 to 2012 (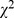 = 12.101, *P*<0.001). All the cases reported in 2011 and 2012 were males (Figure 3-4).

### 3. Outbreaks

During the period of study, only six outbreaks were reported in 2005. Two outbreaks were reported in areas where the criterion of transmission interruption was achieved, namely Xide County, Sichuan province, and Qichun County, Hubei province, respectively. Another four outbreaks occurred in the lake and marshland regions of Hubei province. A total of 55 cases were reported in these outbreaks, and most cases reported they got the infection through swimming (4/6, 66.67%) and farming (2/6, 33.33%).

### 4. Spatiotemporal cluster analyses

A space-time database with 993 cases was established by space-time clustering analysis. The cases in 2011 were excluded from the database because only two cases remained by the pre-processing criterion. Acute infection from 2005 to 2010 presented the similar time frame clustering, with a significant core of clusters from June to November every year (P<0.01). However, the time frame clustered in a limited frame in 2012 with a few cases (Table 4).

**Table 4:** Temporal clustering of acute cases infected with *S. japonicum* with moving window from 2005 to 2010 and 2012.

Year	Time frame	Total number of cases	No. Cases observed	No. Cases expected	Relative risk	Log-likelihood ratio	*P* value
2005	6/12–11/19	546	529	241.28	37.24	364.30	<0.001
2006	6/19–10/22	199	177	68.70	15.26	128.39	<0.001
2007	6/19–10/29	80	71	29.15	13.76	47.62	<0.001
2008	6/26–12/3	53	51	23.31	32.47	34.53	<0.001
2009	6/5–12/3	66	62	32.91	15.59	30.82	<0.001
2010	6/5–11/5	39	39	16.45	infinity	33.65	<0.001
2012	7/31–10/29	10	10	2.49	infinity	13.92	<0.001

A total of 13 cluster regions in space were detected by SaT Scan. However, with the decrease of the reported cases, four clusters did not present significantly different results in 2008, 2010 and 2012, respectively (Table 5). Most of the cluster regions (11/13) were found to overlap in the lake and marshland regions along the Yangtze River Basin, and the other two clusters were found in mountain regions. One of the clusters in 2005 showed the highest Log-likelihood ratio (LLR = 126.56) within the biggest cluster region around the Dongting Lake and Poyang Lake regions in Hunan, Hubei, Jiangxi, and Anhui provinces, which also shows the biggest cluster region overlapped with other clusters in subsequent years. The second cluster in 2005 also showed the highest relative risk value (RR = 69.54) and that only happened in two counties within one week, where the outbreak of Xide County (Sichuan province) happened. Then the LLR decreased over the years with smaller cluster regions. In contrast, however, one of the clusters in 2009 showed the highest relative risk value (RR = 13.44), focused in most parts of Anhui province, then followed by the one of cluster in 2010 (RR = 11) focused in parts of Jiangxi, Anhui and parts of Hubei (Figure 4).

**Table 5:** Spatial clustering of acute cases infected with *S. japonicum* with moving window from 2005 to 2010 and 2012.

Year	Coordinates/radius	Time frame	No. Counties in clusters	No. cases observed	No. cases expected	Relative risk	Log- likelihood ratio	*P* value
2005	30.6457 N, 114.3379 E/240.32 km	7/3–10/1	45	203	61.61	4.87	126.56	<0.001
	27.8532 N, 102.1068 E/49.42 km	7/31–8/6	2	16	0.34	69.54	48.84	<0.001
	25.6921 N, 100.2115 E/52.05 km	6/19–8/13	3	12	2.30	6.60	11.23	<0.01
2006	30.8587 N, 117.3730 E/249.67 km	7/3–9/24	34	82	19.53	6.44	67.60	<0.001
2007	29.8253 N, 115.1297 E/169.14 km	8/7–10/22	18	28	5.63	7.11	26.30	<0.001
2008	30.6426 N, 113.1166 E/379.82 km	7/17–11/12	21	27	6.17	7.89	24.57	<0.001
	31.4983N, 118.6184 E/153.06 km	10/9–11/5	9	8	1.15	8.02	9.15	0.078*
2009	29.8253 N, 115.1298 E/201.08 km	7/17–12/10	18	28	7.84	5.47	19.47	<0.001
	31.4983 N, 118.6183 E/153.06 km	7/24–9/17	9	10	0.87	13.44	16.01	<0.001
2010	30.2405 N, 116.6831 E/317.77 km	7/31–10/15	17	20	3.41	11.00	23.48	<0.001
	29.0120 N, 111.7504 E/74.74 km	7/10–11/5	3	12	3.49	4.52	7.43	0.25*
2012	28.7552 N, 118.1596 E/0 km	8/14–10/29	1	3	0.74	5.35	2.25	0.986*
	30.8587 N, 117.3730 E/136.80 km	8/28–10/1	2	2	0.36	6.69	1.94	0.986*

* *P*>0.05.

## Discussion

Since a re-emergence of schistosomiasis occurred in the early 21^st^ century in P.R. China, the national schistosomiasis control program has been given serious priority in the health agenda in P.R. China [3]. Consequently, the national medium and long-term strategic work plan (2004–2015) for schistosomiasis control was issued in 2004 [28] and the disease control measures were strengthened by using the integrated strategy of control of infection sources after the key role of livestock as a major source of infection was recognized [4]. As a result of these integrated measures achievements have been made in recent years, and the national human and livestock incidence was reduced from 3–5% to 1.70% and 1.38% at administrative village level by the end of 2008, respectively [29]. The mid-term goal of the national plan for schistosomiasis control was achieved by 2009, and the criteria of infection control in all endemic areas and transmission control in mountain areas was reached in Sichuan province in 2008 followed by Yunnan province in 2009 [29]. In 2010 Jiangsu province reached the criteria of transmission control ahead of schedule [30]. The latest data suggest that there were a total of 286,824 cases of schistosomiasis japonica in 2011, a reduction of 65.98% in comparison with 2003, and no occurrence of acute schistosomiasis outbreak had being reported since 2006 [17]. The general endemic situation of schistosomiasis in P.R. China has now reached a historically low level.

The trend of acute human infections of *S. japonicum*, an important indicator in monitoring the national program of schistosomiasis control and risk assessment, from 2005 to 2012, was analyzed in this study. By 2012 acute infections had decreased significantly, with a reduction of 97.7% in comparison with that of 2005. Except for two sporadic- and micro-rebounds in 2009 and 2012, no serious outbreak was reported since 2006. According to the data from 1999 to 2004, three stages were identified after the longitudinal observation on the annual incidence of acute schistosomiasis. The first is that of increasing infection from 1999 to 2003 with a peak of 1,114 acute schistosomiasis cases in 2003 [31]. The available data suggests that schistosomiasis had re-emerged, probably due to multiple factors, including increased snail diffusion after major flooding events and somewhat reduced control efforts when the World Bank Loan Project on schistosomiasis control in China ceased [32], [33]. An estimated 843,011 people were infected with *S. japonicum* during 2003. Among them were 1,114 cases with acute schistosomiasis and 24,441 cases suffering from advanced schistosomiasis. The population at risk of infection was estimated around 65 million and there were 74,000 infected cattle [1].

The second stage is one of decreasing infection rates from 2004 to 2008 with an 89.89% reduction compared with that of 2005 [4]. The priority given to schistosomiasis control after re-defining re-emergence of the disease, led to the implementation of a new comprehensive strategy for schistosomiasis control by the central and local government authorities in the country [5]. By the end of 2008, all of the endemic areas reached the criterion of infection control, with Sichuan and Yunnan provinces reaching the criterion of transmission control in 2008 and 2009 respectively [29]. The number of reported cases of acute human schistosomiasis also declined annually from 564 to 57 [4].

The third stage can be identified as a relative plateau from 2009 to 2012 with unstable rebounds and the lowest number of cases in 2011(n = 3). It suggests that the trend of re-emergence of schistosomiasis during 2000–2003 ceased after implementation of intensive control efforts in a revised strategy initiated late in 2004. It also indicated that the decrease trend of the number of acute schistosomiasis was mainly attributed to the intensified efforts of the new integrated strategy in the national schistosomiasis control program in recent years [5], [34].

Although the total number of acute cases decreased significantly in the past 4 years, without an obvious change in the natural and social factors in the endemic areas, it seems that the situation of human acute schistosomiasis has entered an unstable plateau stage with a high risk of infection remaining in traditional endemic areas [35]. In this study a retrospective space-time analysis identified the lake and marshland regions as susceptible to high-risk of disease and interruption areas being at risk of re-emergence. Nine risk clusters in space and time were detected (P<0.01) by SaT Scan soft, most of which are located in the lake and marshland regions and the middle and lower Yangtze River basin where the environment is favorable for vector snail survival. The risk cluster regions were similar to the results of observed risk areas reveled by the sentinel mice monitoring carried out in high-risk water regions in 2010 [36]. One explanation for those risks remained is that the major source of infection is the bovines in the large marshland areas, especially water buffaloes that the farmers raise and use for their agricultural practices [37]. The pastures used by the animals cannot be isolated easily. Another potential reason is that effort in schistosomiasis control was reduced after the points of transmission control or interruption had been reached [4], [35]. Most of the cases are students, farmers and fishermen who got the infection mainly by swimming in water bodies where transmission and other activities were ongoing during the 27^th^–43^rd^ week every year, and the proportion of imported cases of acute schistosomiasis increased sharply from 2011, which agrees with earlier investigations [4].

This study provides evidence-based information on the importance of current control activities for controlling the risk of acute infection among school-aged children, farmers and fishermen, and especially for primary school-aged students [38]. More importantly, it should help in further evaluation of the effects of schistosomiasis control strategies and the shift of such strategies from control of sources of infection to surveillance and response. The latter could include the setting up of more surveillance sentinel sites in the different endemic areas, establishing networks of diagnosis reference laboratory and capacity building. Moreover, the surveillance capacity should be further strengthened not only in the endemic areas, but also in the non-endemic or transmission-interrupted areas [39], due to mass rural-urban migration movements for socio-economic reasons [40].

In conclusion, the results of this study showed the number or incidence of acute schistosomiasis infections decreased significantly from 2005 to 2012, as shown in the results of this study, indicating that locally ongoing transmission of schistosomiasis has been reduced to a remarkable extent. It also indicates that the integrated strategy of schistosomiasis control has played a key role in reduction of disease burden, both in morbidity-control and infection-control stages. Thus comparatively new cases of schistosomiasis do occur and a potential risk of the acute infection still exists in the lake and marshland regions or transmission-interruption areas. Therefore, there remains a strong need for implementation of intensive surveillance and response activities during the transition stage from control to elimination of schistosomiasis in P.R. China.
